# Ultrafast optical excitation of magnetic skyrmions

**DOI:** 10.1038/srep09552

**Published:** 2015-04-24

**Authors:** N. Ogawa, S. Seki, Y. Tokura

**Affiliations:** 1 RIKEN Center for Emergent Matter Science (CEMS), Wako, Saitama 351-0198, Japan; 2 PRESTO, Japan Science and Technology Agency, Tokyo 102-0075, Japan; 3 Department of Applied Physics and Quantum Phase Electronics Center (QPEC), University of Tokyo, Tokyo 113-8656, Japan

## Abstract

Magnetic skyrmions in an insulating chiral magnet Cu_2_OSeO_3_ were studied by all-optical spin wave spectroscopy. The spins in the conical and skyrmion phases were excited by the impulsive magnetic field from the inverse-Faraday effect, and resultant spin dynamics were detected by using time-resolved magneto-optics. Clear dispersions of the helimagnon were observed, which is accompanied by a distinct transition into the skyrmion phase, by sweeping temperature and magnetic field. In addition to the collective excitations of skyrmions, i.e., rotation and breathing modes, several spin precession modes were identified, which would be specific to optical excitation. The ultrafast, nonthermal, and local excitation of the spin systems by photons would lead to the efficient manipulation of nano-magnetic structures.

Magnetic skyrmion is a nano-scale spin-swirling object protected by topology, discovered to deliver emergent electromagnetic interactions for various potential applications^1^,^2^. After the experimental verification of its existence in the momentum^3^ and in the real space^4^, there have been a variety of techniques proven to be effective to manipulate the skyrmions, including charge and spin currents^5^,^6^, electric field^7^, heat^8^ and magnon^9^,^10^,^11^ to name a few, in a wide range of chiral magnets^1^. Now, spatially-resolved, three-dimensional, and dynamical measurements are becoming crucial for the study of skyrmions, for instance, to realize one-to-one manipulation and to identify magnetic monopoles and antiskyrmions expected to appear in their generation/annihilation processes^12^.

Cu_2_OSeO_3_ is a helimagnet with the noncentrosymmetric *P*2_1_3 structure, and is a unique insulating crystal known to host skyrmions with the Dzyaloshinskii-Moriya interaction^13^,^14^. The skyrmion crystal phase (SkX) appears just below the magnetic transition temperature (~59 K, Fig. 1) with the skyrmion diameter of about 60 nm. In the SkX phase, an emergent magnetoelectric nature, i.e., spin-derived multiferroic effects and nonreciprocal directional dichroism, has also been predicted^15^ and demonstrated^16^,^17^, attracting growing attentions. Especially, collective dynamics at microwave frequencies^18^ are of particular importance, because it will form the bases of the functions of skyrmion memory and oscillator devices. Numerical studies with the two-dimensional Landau-Lifshitz-Gilbert equation have revealed three low-energy collective spin excitations in the SkX phase^9^; two rotation modes with the out-of-plane spin components rotate around the skyrmion core clockwise (CW, higher in energy) or counter-clockwise (CCW, lower) under the in-plane driving ac magnetic field, and a breathing mode under the out-of-plane one. These rotation and breathing modes have been experimentally demonstrated^17^,^18^.

To further control the skyrmions in local (individual) and ultrafast manner, it is promising to utilize optical techniques, since single skyrmions have already been created by pulsed laser excitation in the past^19^,^20^. It is well known that one can apply an effective magnetic field of subpico-second duration by the impulsive Raman process of a circularly-polarized light, so called inverse Faraday effect^21^. This effective field is along the light *k*-vector in the solid, with its amplitude related to the electronic structure and spin-orbit interactions. For the case of insulating samples, one can avoid thermal excitations by using the photons with the energy below the optical gap, thus enabling pure spin excitations. By focusing or structuring the light spot, the spin excitation can be spatially-controlled^22^, possibly down to sub-micron scale^19^,^20^. Therefore it is interesting to perform all-optical spatio-temporal spin wave spectroscopy on the skyrmion systems.

In this report, we demonstrate inverse-Faraday excitation of the collective spin dynamics in Cu_2_OSeO_3_ as a first step. It is found that the non-thermal optical pulse leads to the excitations of helimagnons and rotation/breathing of the skyrmions. Clear beating features in the spin precession signals revealed several fundamental spin wave modes, with mode-dependent decay constants, which are compared to those found in the microwave resonance experiments^17^,^18^. We also identified precession signals not observed in the previous reports, which would be explained by the strong impulsive excitation of the spin system.

## Results and Discussion

**Figure 1:**
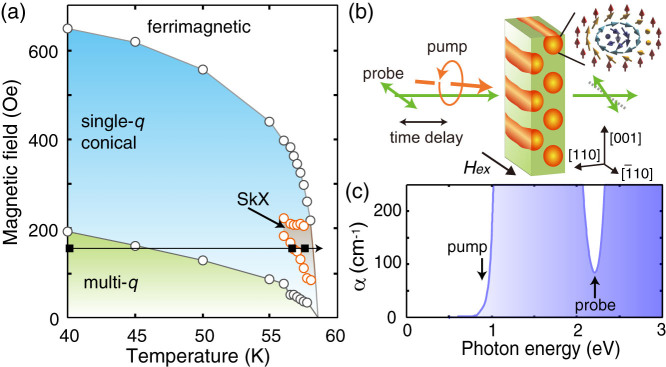
Phase diagram and experimental setups. (a) Phase diagram of a Cu_2_OSeO_3_ single crystal deduced from magnetic susceptibility measurements under the in-plane magnetic field (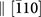). Representative data points shown in Figs. 2 and 3 are indicated by an arrow and solid squares. (b) Schematic optical setup for detecting transient Faraday rotation of the probe light induced by the inverse-Faraday effect of the circularly-polarized pump light. (c) Absorption coefficient of the Cu_2_OSeO_3_ sample with the pump and probe photon energies indicated by arrows.

**Figure 2:**
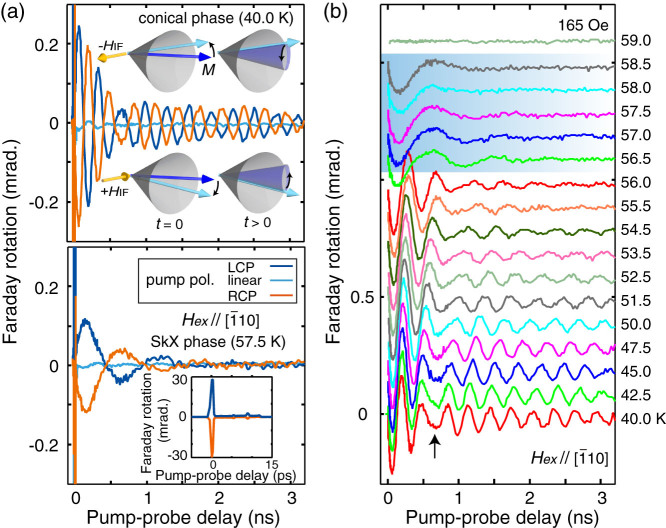
Spin dynamics in the conical and SkX phases. (a) Transient Faraday rotation of the probe light induced by the inverse-Faraday excitation in the conical (upper panel) and the SkX (lower panel) phases under the in-plane magnetic field of 165 Oe (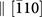). The pump polarization was varied from right-circular (RCP), linear, to left circular (LCP). Insets show the schematics of spin precession and the magnified spectra near the time zero. (b) Temperature evolution of the collective spin dynamics excited by the RCP pump pulse (offset for clarity). The SkX phase is indicated by a shadowed box, and the beating structures in the spectra are indicated by an arrow. The response at time zero is omitted.

The magnetic phase diagram of Cu_2_OSeO_3_ and experimental setups are shown in Fig. 1 (see Methods for the detail). Figure 2 shows the representative spin precession spectra in the conical and SkX phases under the *H_ex_* 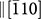 (normal to the photon *k*-vector) [Fig. 1(b)]. In both phases, transient Faraday rotations with opposite initial phase (π-rad. shifted) were observed for right- and left-circular polarized pump excitations (RCP and LCP), respectively. The spin motion was negligible when excited with linearly-polarized pump pulses [Fig. 2(a)], therefore we can safely rule out thermal effects and optically-induced changes in the magnetic anisotropy^23^. We observed no pump-induced change in the probe transmittance (not shown). The spins in the Cu_2_OSeO_3_ feel strong inverse-Faraday field (±*H_IF_*) along the light *k*-vector of the pump pulse [Fig. 2(a) bottom inset], which tilts the magnetic moment (*M*) in the plane normal to the *k*-vector within the pump duration, triggering the spin precession generally expressed as 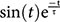 [Fig. 2(a) inset], where *τ* is the decay constant. When the temperature was scanned from 40 to 59 K [see Fig. 1(a)] under the *H_ex_* of 165 Oe, a dispersion of the collective spin dynamics and a distinct transition to the SkX phase was observed as shown in Fig. 2(b). The large Faraday rotation at time zero is omitted from the data in the following.

**Figure 3:**
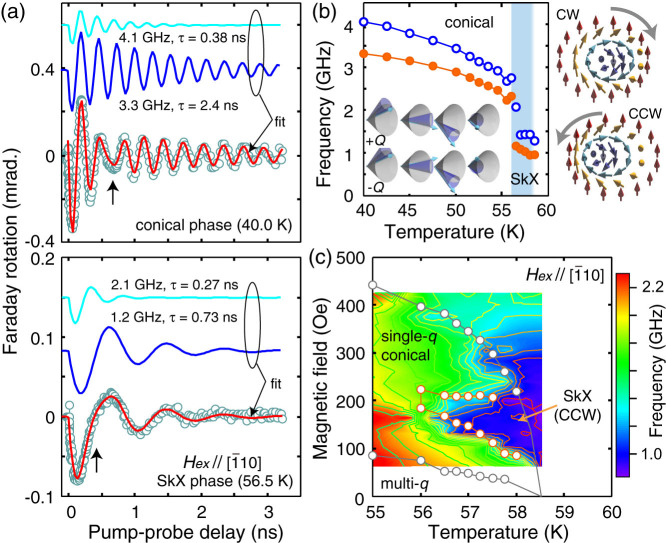
Spin modes under the in-plane magnetic field. (a) Two-component fittings of the transient Faraday rotation spectra under the in-plane magnetic field of 165 Oe (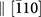) for the conical (upper panel) and SkX (lower panel) phases. The experimental data (open circles) are expressed by the sum of two spin precession modes (blue and light-blue lines) with exponential decay. Beating and shoulder structures are indicated by vertical arrows. (b) Spin precession frequencies extracted by the fitting. Open circles represent +*Q* mode and CW rotation mode, and filled circles −*Q* mode and CCW rotation mode. The error bars are smaller than the markers. Corresponding spin dynamics are also shown schematically. (c) Contour map of the spin precession frequencies plotted together with the phase boundaries deduced from the magnetic susceptibility measurements (open circles).

It is notable that the spin precessions cannot be expressed with a single sinusoidal wave with an exponential decay [a characteristic modulation, called beating below, is exemplified by an arrow in Fig. 2(b)]. This is naturally expected because the conical spins exhibit two fundamental excitation modes in our experimental geometry, called ±*Q* helimagnons^24^, which have been detected in Cu_2_OSeO_3_ with the microwave resonance spectroscopy^17^,^18^. We also found that the spin precession in the SkX phase cannot be expressed with a single frequency. Therefore, we fitted all the spectra with two sinusoidal functions, as shown in Fig. 3(a), which allowed us to avoid arbitrary determination of the phase boundaries. (In the lower panel of Fig. 3(a), the spectrum near the phase boundary with a clear shoulder structure is shown.) The fitting works reasonably well in both conical and SkX phases, yielding temperature- and field-dependent oscillation frequencies, amplitudes, and damping constants. The oscillation frequencies are plotted as a function of temperature in Fig. 3(b), showing a discontinuous transition from the conical phase into the SkX phase. By comparing to the previous reports^17^,^18^, we can assign the observed spin dynamics to the ±*Q* helimagnons in the conical phase and rotation modes of the skyrmions [Fig. 3(b) insets], as will be discussed in detail below. By rendering the oscillation frequencies (of the −*Q* mode and the CCW rotation) in the phase diagram, we can nicely visualize the SkX phase [Fig. 3(c)].

**Figure 4:**
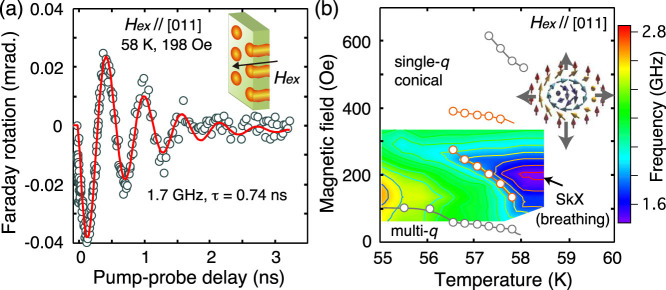
Spin mode under the out-of-plane magnetic field. (a) Transient Faraday rotation of the probe light with the external magnetic field applied along the [110] axis (nearly parallel to the photon *k* vector). The red line shows the fit with a single spin precession with exponential decay. (b) Contour map of the spin precession frequencies plotted together with the phase boundaries deduced from the magnetic susceptibility (open circles).

When the external magnetic field *H_ex_* is applied along the sample normal (|| [110], Fig. 4), only one collective spin mode is expected in the SkX phase (and no mode in the other phases)^9^. In this setup, the Faraday rotation of the probe light is smaller than that of the former setup, consistent with the previous report^18^. We found that the observed spin dynamics can be fitted with a single sinusoidal function [Fig. 4(a)]. The deduced oscillation frequencies fell into those of the skyrmion breathing motion, which also nicely reproduce the phase diagram deduced from the magnetic susceptibility measurements [Fig. 4(b)].

The oscillation frequencies of the helimagnons, and those of the rotation and the breathing modes in the SkX phases nicely match to the values found in the previous reports^17^,^18^. Judging from this fact and also from the phase maps in Figs. 3(c) and 4(b), it is concluded that we have successfully detected skyrmions from their optically-excited dynamics. We note that the CW rotation mode has been inferred only from the nonreciprocal directional dichroism^17^, due probably to the small spectral weight as expected from the numerical calculations^9^. In contrast, we clearly identified two spin precessions in the SkX phase as shown in Fig. 3. There, the high frequency mode (~1.5 GHz) is not due to the mixing of the conical phase, judging from the discontinuous transition from the conical phase to the SkX phase seen in Fig. 3(b).

**Figure 5:**
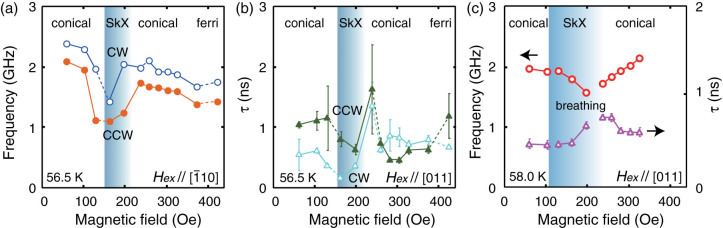
Spin dynamics as a function of external magnetic field. Spin precession frequencies and decay constants as a function of the external magnetic field, for (a),(b) 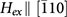 and (c) *H_ex_*∥[110].

To scrutinize the observed spin modes, we plot the magnetic-field dependence of the spin precessions in Fig. 5 for the different magnetic-field orientations. For the *H_ex_* 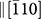 [Fig. 5(a)], the precession frequency decreases with increasing the *H_ex_* in the conical spin phase^24^, whereas increases with increasing *H_ex_* in the SkX phase. For the *H_ex_*||[110] [Fig. 5(c)], the frequency decreases in the SkX phase. These observations are consistent with the expected behaviors of the conical spin phase, rotation modes and the breathing mode of the skyemions, respectively^17^. Therefore, in addtion to the reasoning from its absolute frequency, the higher-lying mode in the SkX phase can be assigned to the CW rotation. The reentrant behavior of the conical phase by increasing the *H_ex_*, after experiencing a pocket of the SkX phase at 56.5 K [Fig. 5(a)], also supports the identification of the SkX phase, since this pocket is well isolated from other spin phases. It is also expected that the damping increases in the SkX phase^25^, which is roughly captured in our data in Fig. 5(b). The long-lasting spin precession at low temperatures [Fig. 2(b)] indicate that the spin scattering is mainly from thermal agitations.

The optical excitation can be distinct from that exerted by the microwaves, because the pump light imposes a strong impulsive magnetic-field at localized region (~100 *μ*m*φ* in our case), possibly with spatial gradients, thereby enabling the coupling to the spin waves with a finite range of frequencies and wave numbers. The amplitude of the impulsive magnetic-field would reach to several thousand Oe^23^, much larger than the external *H_ex_* of the current experiment [see also the inset of Fig. 2(a)] and the ac field of the previous microwave resonance experiments^17^,^18^. In such a situation, multi-magnon processes and the parallel pumping of the magnons (for *H_ex_* ||[110]) are expected. The latter generates two magnons of opposite wavevectors (±*k* ≠ 0) by the ellipticity of the precession orbit^26^. We tentatively ascribe the precession mode for *H_ex_* > 220 Oe ||[110] [Fig. 5(c)] to these excitations. It will be necessary to consider the dispersion relation of the collective spin modes for the non-zero *k*, as has been discussed in the case of magnetic bubble lattices^27^, especially if the excitation light spot close to the skyrmion size is employed (not the case here). The spin precessions seen in the low *H_ex_* region can be ascribed to the multi-domain states, while those in the ferrimagnetic phase (the high *H_ex_* region 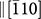) to the ferromagnetic and exchange modes having similar frequencies near the transition temperature^28^. Since the spins in Cu_2_OSeO_3_ are found to form a quantum triplet states within the Cu_4_ tetrahedra^29^,^30^, these spin dynamics would be better described by incorporating the quantum fluctuations. We note that the optical excitation can also generate “forbidden” spin resonances by using site-dependent magneto-optical susceptibilities^31^.

There is one feature to be noted and explained; the spin precession seems excited in the intermediate phase, i.e., slightly above the magnetic transition temperature [around 58.5 K and 300 Oe in Fig. 3(c)]. The intermediate phase has larger spin fluctuations^14^, expected to host isolated skyrmions or fragmented skyrmion domains, and to be susceptible to the magnetic history of the sample. The spin dynamics invoked in this region may indicate the existence of these fragmented domains^19^,^20^, or other induced spin excitation by the strong inverse-Faraday field.

In the metallic helimagnet Fe_0.8_Co_0.2_Si, which is also known to host skyrmions, the spin dynamics has been analyzed with time-dependent Gilbert dampings^32^, since no beating features in the precession signals were detected by the optical spectroscopy. In the case of Cu_2_OSeO_3_ here, the beating and shoulder structures in the spin signals revealed the distinct multiple precession dynamics.

## Conclusions

To summarize, we have successfully detected the collective excitation modes of skyrmions in the insulating chiral magnet Cu_2_OSeO_3_ by optical pump-probe techniques. The rotation and breathing modes are identified in the skyrmion phase, and several additional spin precession modes have also been detected. We discussed the difference between the inverse-Faraday and microwave excitations, which may explain the observed multi-mode spin excitations.

## Methods

Single crystals of Cu_2_OSeO_3_ were grown by the chemical vapor transport method, and were polished down to 300 *μ*m in thickness to expose (110) planes. The conical spin and SkX phases were checked by the magnetic susceptibility measurements [Fig. 1(a)], in which some demagnetization effects make small deviations from sample to sample^18^. We studied the spin dynamics by the time-resolved magneto-optics with pulsed laser sources (~120 fs, 1 kHz) at near-normal incidence [Fig. 1(b)]. The pump pulse (2 mW on ~100 *μ*m*φ* spot) excite the spin system at 0.95 eV (within the optical gap) by the inverse-Faraday effect, and the induced spin precession was detected by the Faraday rotation of the linearly-polarized probe pulse at 2.2 eV [Figs. 1(b) and (c)]. Thus the probe pulse detects the change in the out-of-plane (|| [110]) component of the magnetization. Note that the pump pulse at 0.83 eV induced qualitatively the same spin dynamics with slightly reduced efficiency (not shown). The external magnetic field (*H_ex_*) was applied by a pair of permanent magnets, which limited the phase space studied in our experiment.
